# Open-source image reconstruction of super-resolution structured illumination microscopy data in ImageJ

**DOI:** 10.1038/ncomms10980

**Published:** 2016-03-21

**Authors:** Marcel Müller, Viola Mönkemöller, Simon Hennig, Wolfgang Hübner, Thomas Huser

**Affiliations:** 1 Biomolecular Photonics, Department of Physics, University of Bielefeld, 33615 Bielefeld, Germany; 2 Department of Internal Medicine and NSF Center for Biophotonics, University of California, Davis, Sacramento, California 95817, USA

## Abstract

Super-resolved structured illumination microscopy (SR-SIM) is an important tool for fluorescence microscopy. SR-SIM microscopes perform multiple image acquisitions with varying illumination patterns, and reconstruct them to a super-resolved image. In its most frequent, linear implementation, SR-SIM doubles the spatial resolution. The reconstruction is performed numerically on the acquired wide-field image data, and thus relies on a software implementation of specific SR-SIM image reconstruction algorithms. We present fairSIM, an easy-to-use plugin that provides SR-SIM reconstructions for a wide range of SR-SIM platforms directly within ImageJ. For research groups developing their own implementations of super-resolution structured illumination microscopy, fairSIM takes away the hurdle of generating yet another implementation of the reconstruction algorithm. For users of commercial microscopes, it offers an additional, in-depth analysis option for their data independent of specific operating systems. As a modular, open-source solution, fairSIM can easily be adapted, automated and extended as the field of SR-SIM progresses.

## Abstract (web summary)

Reconstruction of super resolution structured illumination microscopy (SR-SIM) datasets typically relies upon commercial software. Here Müller *et al.* present an open-source ImageJ plugin to facilitate reconstruction of SR-SIM data from a broad range of microscopy platforms.

## Graphical abstract

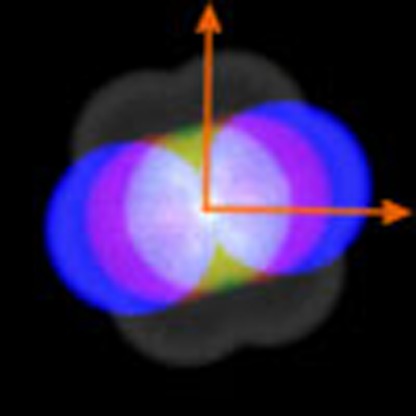

The improvement in spatial resolution achieved in super-resolved structured illumination fluorescence microscopy (SR-SIM) is accomplished by illuminating a sample with a well-defined set of sinusoidal illumination intensity patterns, that is, typically a set of interference patterns^1^. The light modulation leads to frequency mixing between the harmonic pattern frequency and the sample frequencies, which is then demodulated by a digital image reconstruction step^2^. This enables access to previously unobservable high-frequency components, and thus improves spatial resolution. For linear SR-SIM, the illumination pattern adheres to (approximately) the same resolution limit as the imaging path, hence SR-SIM doubles the spatial resolution in comparison with a wide-field image^3^.

The principle and design of the instrumentation for SR-SIM is well documented in the literature^1^,^2^, and the technique is now in wide use^4^,^5^,^6^,^7^,^8^,^9^,^10^. It has also been successfully combined with other optical techniques^11^,^12^,^13^,^14^,^15^,^16^, where non-linear approaches^17^,^18^,^19^,^20^ allow to surpass the factor of 2 × in resolution improvement.

SIM data sets are usually acquired by a modified wide-field microscope, where a light-modulating component is introduced into the excitation path. Nowadays, commercial SR-SIM platforms are available by different manufacturers. Also, spatial light modulators (SLMs) offer a simple, robust and cost-efficient way to custom-build such systems. Recent publications provide detailed blueprints for home-built SR-SIM microscope set-ups^21^,^22^,^23^, focusing on the design of customizable, cost-effective and fast systems.

The algorithm required for SR-SIM reconstructions can readily be found in the literature, for example, in the publication by Gustafsson *et al.*^2^. However, as of now, there is no implementation available for ImageJ. Existing solutions are either provided as proprietary components of commercial SIM microscopes, often bound to a dedicated workstation computer, or exist as purpose-written tools by different research groups^24^. In the related field of super-resolved localization microscopy, the situation is rather different. Various well-known open-source solutions, for example, QuickPALM^25^ and rapidSTORM^26^, with different feature sets (a summary of which was published recently^27^), and often direct integration with ImageJ, are available for data analysis.

This, together with our own need for a SIM reconstruction software, motivated the development of fairSIM (free analysis and interactive reconstruction for structured illumination microscopy). FairSIM is aimed at providing a ready to use, easy to operate, free and open-source solution for SR-SIM. It features a plugin that integrates directly into ImageJ^28^/Fiji^29^, allowing it to use all image formats supported by ImageJ, and easy integration with its other pre- and post-processing steps.

## Results

### Concept and motivation for developing fairSIM

FairSIM was primarily designed to provide single-slice reconstructions of SR-SIM systems working with both two-beam interference (utilized by many home-built and total internal reflection-excited fluorescence (TIRF)-based systems) and three-beam interference (utilized in all commercially available systems) for pattern generation. This development was motivated by the need for both a rapid and flexible single-slice reconstruction mode for our own commercial SR-SIM system^30^, and a stand-alone reconstruction tool for our home-built, two-beam illumination SIM set-ups.

FairSIM is implemented as Java plugin, so it allows to carry out SR-SIM reconstructions directly from within ImageJ/Fiji. It is based on the well-established SIM illumination technique introduced by Gustaffson^1^,^2^ and Heintzmann^31^, and the corresponding reconstruction algorithms. By combining multiple raw images, each acquired under structured illumination by a defined pattern, these algorithms allow to enhance the resolution twofold in comparison with the corresponding wide-field image.

**Figure 1:**
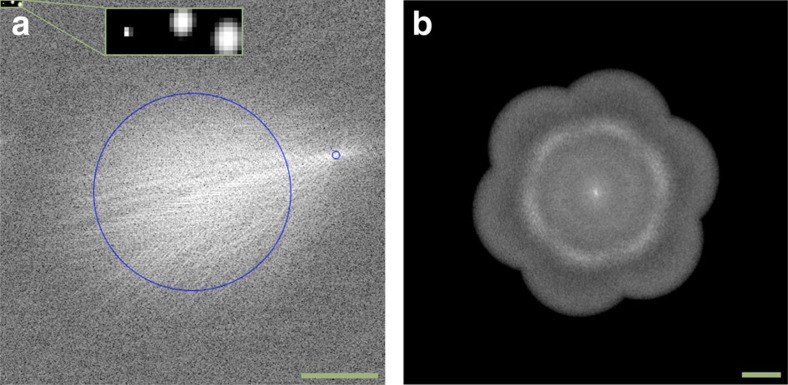
Examples for intermediate SR-SIM results displayed as power spectra in frequency space. (**a**) Visualization of the cross-correlation used for parameter estimation, with circles marking the low-frequency region excluded from the fit and the detected modulation frequency. The three insets on the upper left visualize the iterative sub-pixel fits and provide a quick feedback if the fit was successful. (**b**) Power spectrum of the complete, reassembled SR-SIM reconstruction of fluorescent beads (Fig. 3). The circular structure visible in the spectrum is located at ∼4.5 μm^−1^, which coincides with the beads' size of 200 nm, and is thus expected. Scale bar, 2.5 μm^−1^.

The advantage of single-slice reconstruction of SR-SIM images is that only raw images from one focal plane are needed. Thus, because it is not necessary to acquire multiple image sequences obtained at different vertical focus positions (*z*-stacks), image acquisition is much quicker. This is especially useful for live-cell applications, where short exposure times reduce photodamage and motion blur. As a drawback, however, the axial resolution is not improved. Lateral resolution enhancement is not impaired, and fairSIM retains optical sectioning through optical transfer function (OTF) attenuation^32^,^33^,^34^. Employable especially for three-beam interference data, the attenuation greatly reduces background contributions in thicker samples and thus mitigates reconstruction artefacts (Supplementary Fig. 1). Importantly, for advanced users and in-depth analysis, fairSIM also provides access to various intermediate results of the parameter estimation and reconstruction process in both frequency and spatial domains (Fig. 1). This greatly helps expert users to quickly judge progress and quality of the image reconstruction process, as well as in the analysis of more critical data sets, obtained for example when tuning home-built SR-SIM set-ups.

### Testing fairSIM with different samples and microscopes

We extensively tested the capabilities of fairSIM with SR-SIM data sets acquired on different microscope platforms. A commercial DeltaVision|OMX (GE Healthcare, Issaquah, WA, USA) was used to provide the high-quality three-beam interference illumination data, and a much simpler, home-built, SLM-based two-beam interference illumination system was used to test the compatibility with less refined systems. Furthermore, data sets from systems available by other commercial manufacturers were also tested as described in detail below.

**Figure 2:**
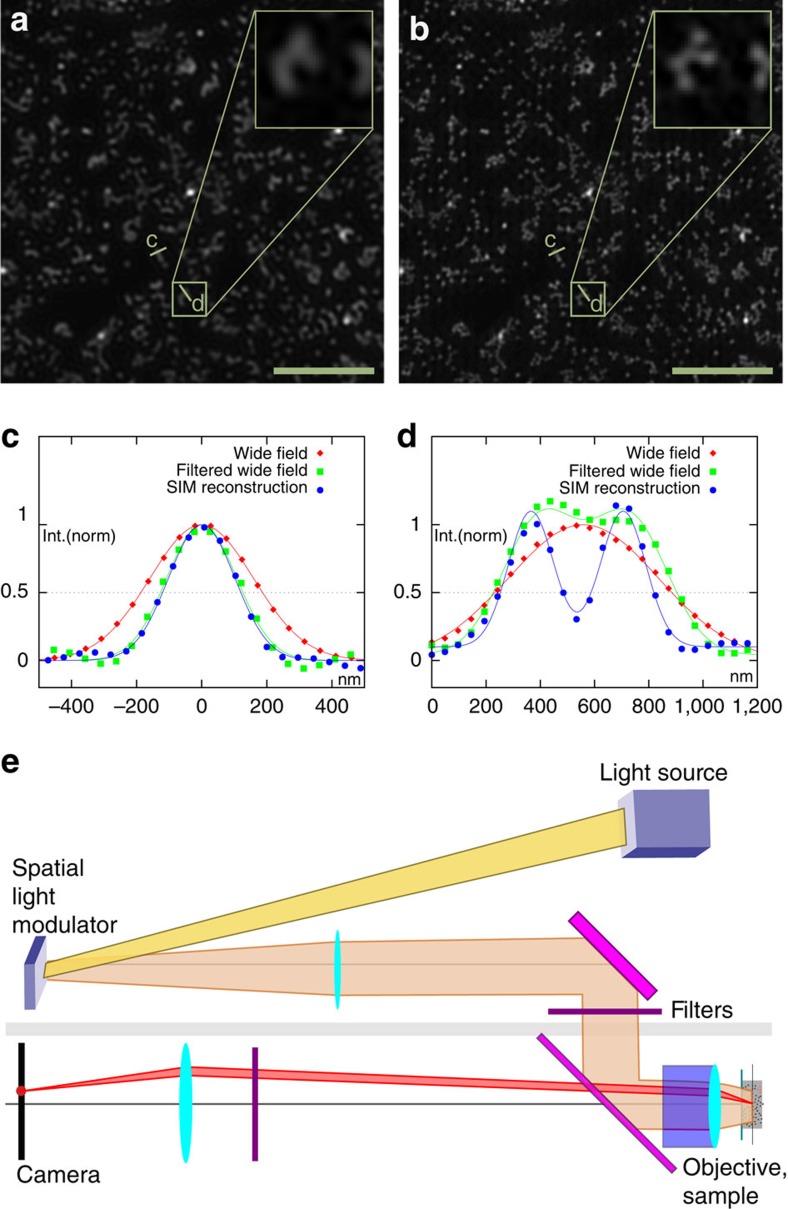
FairSIM reconstruction of data sets obtained on a simple SLM-based SR-SIM setup. A glass surface with 200 nm Tetraspeck beads was used as test sample, excitated at 642 nm wavelength. In contrast to the (Wiener filtered) wide-field image (**a**), the SR-SIM reconstruction by fairSIM (**b**) yields clearly distinct beads. This can be found quantitatively from the cross-section plots, given for a single bead in **c** and two close-by beads (indistinguishable in wide-field mode) in **d**. A simplified sketch of the set-up used is given in **e**. Scale bar, 5 μm, inset 1.2 μm.

**Figure 3:**
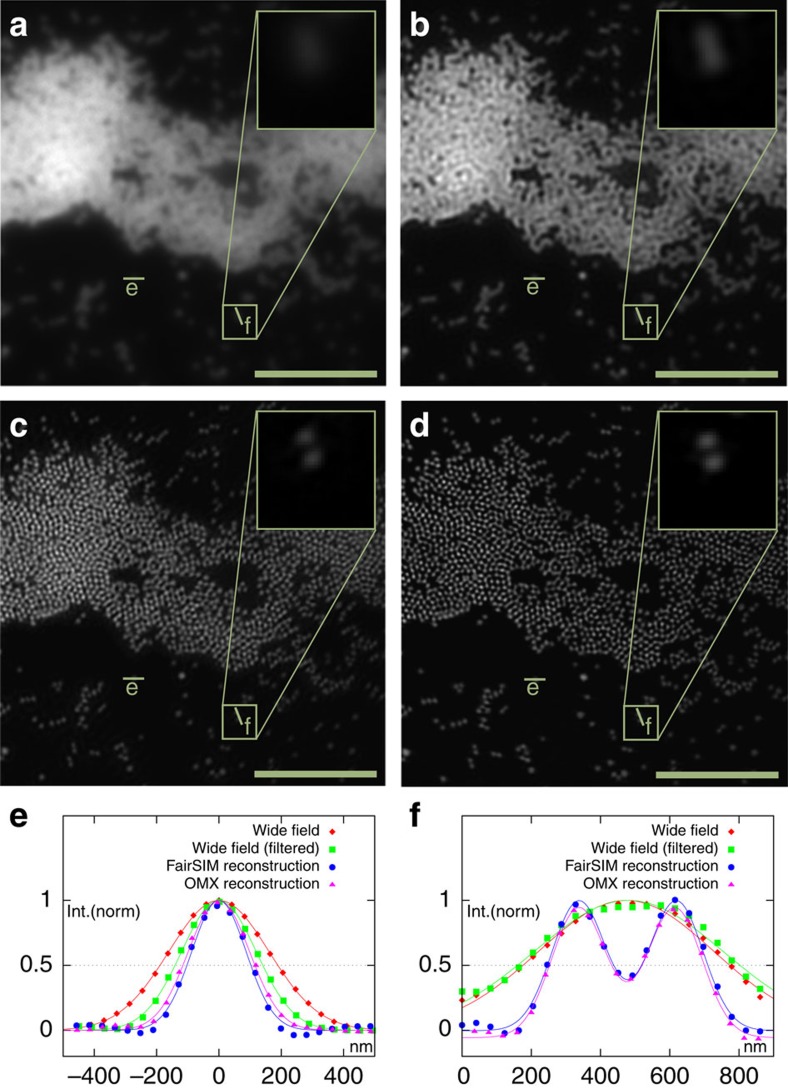
FairSIM reconstruction of data sets obtained on the GE Healthcare DeltaVision|OMX. A glass surface with 200 nm Tetraspeck beads was used as a test sample, excitated at 642 nm wavelength. In contrast to the wide-field image (**a**) and its Wiener-filtered version (**b**), the 2D reconstruction by fairSIM (**c**) yields clearly distinct beads. The 3D SR-SIM reconstruction by SoftWORX (manufacturer's software) is provided in **d** for comparison. Please note that the 3D reconstruction is based on a larger amount of input data (complete *z*-stack), thus resulting in an improved signal-to-noise ratio. A quantitative comparison between all four images can be found as cross-section plots for a single bead in **e** and for two adjacent beads (indistinguishable in wide-field mode) in **f**. Scale bar, 5 μm, inset 1.2 μm.

To characterize SR-SIM set-ups and software, fluorescent bead surfaces have become a standard test sample, as they provide easily quantifiable results. Using a defined bead size slightly below the optical resolution limit, beads in a dense surface cannot be distinguished in a wide-field fluorescence image. Only after applying SR-SIM, and thus improving the resolution twofold, individual beads become distinguishable. We imaged fluorescent Tetraspeck bead surfaces on both our home-built system (Fig. 2) and on the OMX (Fig. 3). In both cases, the beads are only separable in the SR-SIM reconstruction, but not in the wide-field image assembled from the SR-SIM image stack. A comparison with a full three-dimensional (3D) reconstruction is provided for images acquired on the OMX, where the cross-section plots show that both single-slice and full 3D reconstruction achieve a comparable improvement in lateral resolution.

**Figure 4:**
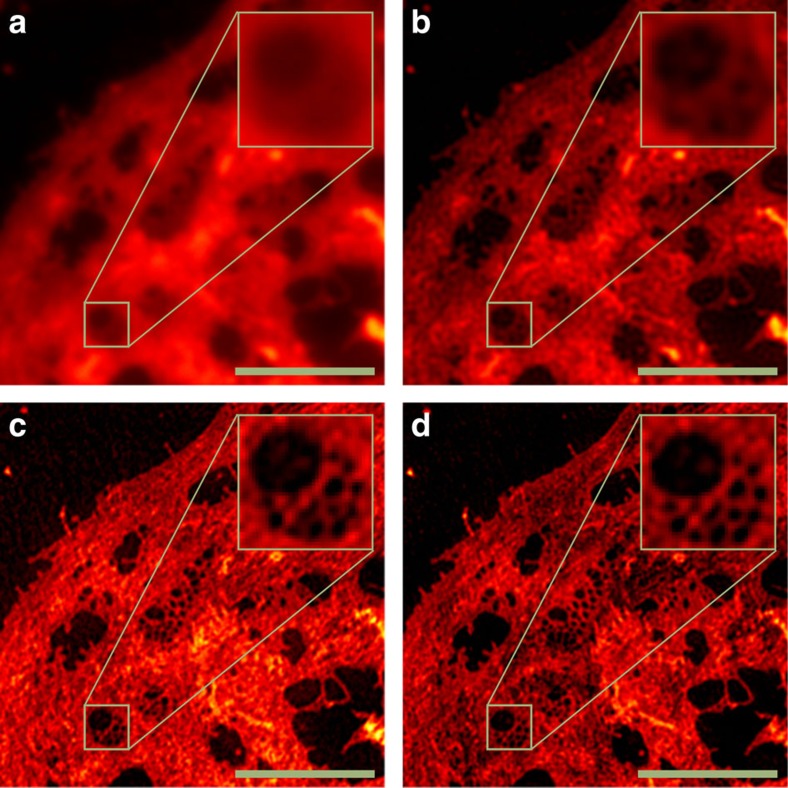
SR-SIM measurement of an LSEC membrane stain obtained on a GE Healthcare DeltaVision|OMX. Wide-field image (**a**), Wiener-filtered wide-field (**b**), single-slice/2D SR-SIM reconstruction by fairSIM (**c**) and full 3D SR-SIM reconstruction by SoftWORX (manufacturer's software) (**d**) are shown for comparison. Both SR-SIM reconstructions allow to clearly identify the cell's fenestrations (tiny membrane pores), which is not possible in the wide-field images. The single-slice reconstructions by fairSIM can be performed with a much lower number of input images, as no *z*-stack has to be acquired. Scale bar, 5 μm, inset 1.6 μm, cells stained with CellMask Deep Red.

As a typical sample encountered in biological applications, liver sinusoidal endothelial cells (LSECs) stained with a fluorophore that selectively stains the plasma membrane of cells (Fig. 4) were imaged on the DeltaVision|OMX and reconstructed both as single slice (fairSIM) images and in full 3D (SoftWORX by DeltaVision). The main structural feature of these rather flat cells, that is, the cellular fenestrations (nano-sized pores that cross the cytoplasm), vary in size between 50 and 200 nm (typically 120 nm)^16^,^36^,^37^,^38^, and are thus ideal candidates for SR-SIM imaging. As with the fluorescent beads, they are too blurred in standard wide-field images, but become clearly visible in both single-slice and full 3D SIM reconstructions. The dynamics of fenestrations in living LSECs is a subject of current research. Therefore, being able to successfully image these submicroscopic structures in single-slice mode (and thus very fast) is of high interest.

As additional tests, other typical cellular structures often imaged by super-resolution optical microscopy methods, namely, cytoskeletal protein fibrils (actin and tubulin) and mitochondria were imaged (Supplementary Figs 2, 3 and 4). Again, comparison between single-slice and full 3D reconstructions demonstrates the validity and usefulness of our plugin.

Data sets of actin filaments and mitochondria, acquired on another commercial SR-SIM system (Elyra S1, Zeiss, Jena, Germany—see Supplementary Figs 5 and 6), were also reconstructed by fairSIM as single-slice images and by the manufacturer software in full 3D and further demonstrate the compatibility with other commercial implementations. A TIRF SR-SIM data set of tubulin fibres (Supplementary Fig. 7), acquired on the set-up built and used by Kner *et al.*^5^, shows the compatibility with other, advanced custom-built SR-SIM systems.

### Compatibility with different SR-SIM microscopes

FairSIM has been developed with a focus on compatibility with a wide range of experimental SR-SIM implementations. Besides providing support for both two-beam and three-beam interference-based illumination, SR-SIM datasets can be acquired with any reasonable number of pattern orientations and phases. Thus, fairSIM is able to handle data sets from all SR-SIM microscope platforms using standard sinusoidal illumination patterns as introduced by Gustafsson^1^,^2^, including the currently available commercial platforms (GE Healthcare, Zeiss, Nikon) and the now popular custom-built setups.

Precise knowledge of the SR-SIM acquisition parameters (pattern orientation, frequency and phases) is required for a successful image reconstruction. Indeed, most often reliable parameter estimation is much more involved than the image reconstruction process itself. State-of-the-art parameter estimation employs weighted cross-correlation of frequency components to obtain these parameters^2^,^32^,^39^. By default, fairSIM uses the Gustafsson approach^2^ (briefly described in Supplementary Note 1 and visualized in Supplementary Fig. 8) to obtain reconstruction parameters, and also features an implementation of a more current phase-optimization algorithm^32^.

### Automated reconstruction parameter estimation

For data sets of adequate quality, fairSIM offers a largely automated mode of operation. Here the user selects the raw images for reconstruction and provides basic parameters of the microscope optics and acquisition mode, that is, the number of bands (two- or three-beam interference illumination), the number of SR-SIM pattern orientations, the number of phases used in the illumination, the effective pixel size and order of the image sequence. For the commercial GE Healthcare DeltaVision|OMX and the Zeiss Elyra systems, presets are available. Also, an OTF, matching the microscope and the emission wavelength, has to be set. Ideally, the OTF has been measured experimentally, and can be read in from file. Alternatively, a basic estimation based on the numerical aperture and main emission wavelength of the fluorophores used in the sample is available.

After this initial set-up and raw data import step, the parameter estimation extracts pattern orientation, angle and phase from the data automatically via cross-correlation (Supplementary Note 1), optionally providing visual feedback to check for plausibility. Obtaining a precise estimate of the SIM reconstruction parameters is a crucial step for successful image reconstruction, because even a small error in these parameters will degrade the quality of the reconstructed image^39^,^40^,^41^. Providing an algorithm that automatically extracts these parameters with little knowledge of the microscope platform in use, without much user interaction and with an easily interpretable feedback was a major point in the development of fairSIM.

In the second step, the actual image reconstruction process is run. As this reconstruction step is much faster than the parameter estimation, it can easily be run multiple times, either to reconstruct multiple images where no changes to the illumination parameters have been made or to tweak image reconstruction filter settings (OTF attenuation, Wiener filter and apodization).

For both the parameter estimation and the image reconstruction steps, different levels of intermediate output (results per band, per pattern orientation, in frequency and spatial domain) can be selected.

### Technical realization of fairSIM

FairSIM features a plugin that readily integrates into Fiji^29^/ImageJ^28^, allowing it to use all image formats supported by ImageJ and seamless integration with other pre- and post-processing steps available in ImageJ. Written entirely in Java, fairSIM supports all computer platforms and operating systems that run ImageJ without much installation effort. FairSIM takes advantage of current multi-core central processing units, running computationally intensive functions in parallel. On a typical desktop computer, that is, using a current-generation quadcore processor, 15 raw images of 512 × 512 pixels reconstruct to a high-resolution image of 1,024 × 1,024 pixels in <3 s. The initial parameter estimation is performed in <20 s, providing all intermediate output takes at most 60 s.

A modular layout, providing defined interfaces for, for example, basic linear algebra, the SR-SIM algorithm, the graphical user interface and the integration with ImageJ, allows one to easily reuse and extend fairSIM's components. Most importantly, low- and high-level functionality of the SR-SIM algorithm module can easily be used through Fiji's scripting facilities, allowing advanced users to run reconstructions without resorting to the graphical user interface, and thus to automate their workflow. Also, data structures and functionality in our linear algebra package can be employed to implement new reconstruction methods, providing much more convenience than working with pure Java data types.

Our source code is freely available under GNU general public license (GPL) and managed on Github. Together with the modular layout, this facilitates the ease of modifications and extensions, for example, towards the reconstruction of non-linear SR-SIM data sets^17^,^18^,^19^,^20^ or to implement novel reconstruction techniques^42^,^43^,^44^,^45^.

## Discussion

We expect that fairSIM will become a highly useful tool for super-resolution structured illumination microscopy. With the current advent of blue prints for cost-effective, fast and customizable SR-SIM microscopes, research groups building these set-ups benefit from a ready-to-use reconstruction software, so they do not have to create their own customized implementation. Our solution is also modular and flexible enough to be adapted to new needs, for example, the automated processing of large time series or the extension to non-linear SR-SIM methods. Users of commercial SR-SIM microscope platforms obtain a tool to perform reconstructions independent of the manufacturer software, which allows in-depth data analysis and is not bound to a specific microscope workstation. FairSIM is freely available as a system-independent, ready-to-use ImageJ plugin and as open-source code. We provide a collection of raw test image sequences, including all raw data sets needed to reproduce the figures presented here, as an additional download for fairSIM. We also provide a short user manual, which includes all parameters needed for these reconstructions.

## Methods

### Access to the plugin and source code

A ready-to-use version of the plugin, the source code, example data sets and a short user manual can be found online. All resources are hosted publicly on github, accessible via http://fairsim.org or https://github.com/fairsim, and can also be reached through our institute website, http://www.bio-photonics.de.

### SR-SIM microscopy systems

Images from two commercially available SIM microscopes were analysed, obtained on a Delta-Vision|OMX v4 by GE Healthcare (Issaquah, WA, USA) and on an Elyra S1 by Zeiss (Jena, Germany). Also, raw images were acquired on a home-built, SLM-based two-beam interference illumination SR-SIM microscope. This system consists of a 60 × , 1.2 numerical aperture water immersion objective (Olympus, Hamburg, Germany), a 642 nm, 85 mW fiber-coupled diode laser for excitation, a charge-coupled device camera (Coolsnap HQ, Photometrics, Tuscon, AZ, USA) and a liquid crystal display-based SLM for light modulation (LC-R 1920, Holoeye Photonics, Berlin, Germany). A sketch of the set-up can be found in Fig. 2e. The TIRF SR-SIM set-up is documented by Kner *et al.*^5^.

### Preparation of TetraSpeck bead surfaces

TetraSpeck microspheres (0.2 μm, T-7280) were purchased from Thermo Fisher (Waltham, MA, USA). Commercially available coverslips (∼150 μm) with 24 × 60 mm in size were carefully cleaned with HelmanexIII (Hellma GmbH Göttingen, Germany) for 20 min in a supersonic bath at 45 °C. Then, the coverslides were rinsed for two times with pure H_2_O, followed by another 20 min in the supersonic bath in pure H_2_O. Then, the coverslides were dried in an air flow prior use. A silicone sheet (self-adhesive; Sigma-Aldrich (GBL666182-5EA)) with a hole of 4 mm in diameter was disposed to the coverslip. A measure of 5 μl stock solution of TetraSpeck Microspheres was mixed with 5 μl H_2_O and vortexed for 2 min. The solution was dispensed onto the coverslip and dried headfirst over night at 4 °C.

### Cells

Rat LSECs were isolated from Sprague Dawley male rats (Scanbur BK, Sollentuna, Sweden) kept and fed under standard conditions. The treatment of the animals was performed in accordance with the Norwegian Animal Experimental and Scientific Purposes Act of 1986. The experimental protocols were approved by the Norwegian National Animal Research Authority (NARA). Young rats with a body weight between 150 and 300 g were killed with a mixture of medetomidin (Domitor vet, Orion, Turku, Finland) and ketamine (Ketalar, Pfizer, New York, NY). The liver was perfused with collagenase and the isolated liver cells were centrifuged on a Percoll density cushion. The fraction containing the LSECS was plated for selective adherence on fibronectin coated #1.5H coverslips (Paul Marienfeld GmbH & Co KG, Germany) for 3 h in RPMI-1640. At 3 h after plating, they were fixed with 4% paraformaldehyde for 15 min. For fluorescent staining of membranes, the LSECs were incubated with CellMask Deep Red plasma membrane stain (Thermo Fisher, #C10046; 1:2,000) for 10 min at room temperature (Fig. 4). LSECs were additionally permeabilized with 0.5% Triton-X for 30 s prior staining with 165 nM phalloidin Atto488 (Sigma Aldrich, #49409) for 20 min at room temperature (Supplementary Fig. 2). Human osteosarcoma cells U2OS (ACC785—DSMZ, Braunschweig, Germany) plated on uncoated #1.5H cover glass were fixed with 4% paraformaldehyde for 10 min. The U2OS cells shown were permeabilized with 0.5% Triton-X100 for 30 s prior staining with 165 nM phalloidin Atto488 (Sigma Aldrich, 49409) for 20 min at room temperature (Supplementary Fig. 1). For additional immunofluorescent staining, U2OS cells were permeabilized for 60 s with 0.5% Triton-X100, then washed in PBS, blocked with a 5% bovine serum albumin in PBS solution for 60 min at room temperature and incubated with 5 μg ml^−1^ of mouse anti-α-tubulin-Alexa488 (Thermo Fisher, #322588) in PBS containing 5% bovine serum albumin for 90 min at room temperature. Prior mounting, the samples were washed three times for 5 min with 0.1% Tween-20 in PBS and once with PBS (Supplementary Fig. 3). All fixed samples were mounted in Vectashield (Vector Laboratories, H-1200) on a glass slide and sealed with nailpolish. In Supplementary Fig. 4, the live U2OS were stained for 30 min with 500 nM MitoTracker Red CM-H2Xros (Thermo Fisher, #M-7513) in DMEM for 30 min at 37 °C and then imaged at room temperature on the Deltavision|OMX.

## Additional information

**How to cite this article:** Müller, M. *et al.* Open-source image reconstruction of super-resolution structured illumination microscopy data in ImageJ. *Nat. Commun.* 7:10980 doi: 10.1038/ncomms10980 (2016).

## Supplementary Material

Supplementary Information Supplementary Figures 1-8, Supplementary Note 1 and Supplementary References
