# Supplementary material for: Open-source image reconstruction of super-resolution structured illumination microscopy data in ImageJ
Source: Nat Commun. 2016 Mar 21;7:10980. doi: 10.1038/ncomms10980 (PMC4802170; doi:10.1038/ncomms10980)
Supplement: Supplementary Information — Supplementary Figures 1-8, Supplementary Note 1 and Supplementary References [file ncomms10980-s1.pdf]

Effect of OTF attenuation on single-slice SR-SIM reconstruction

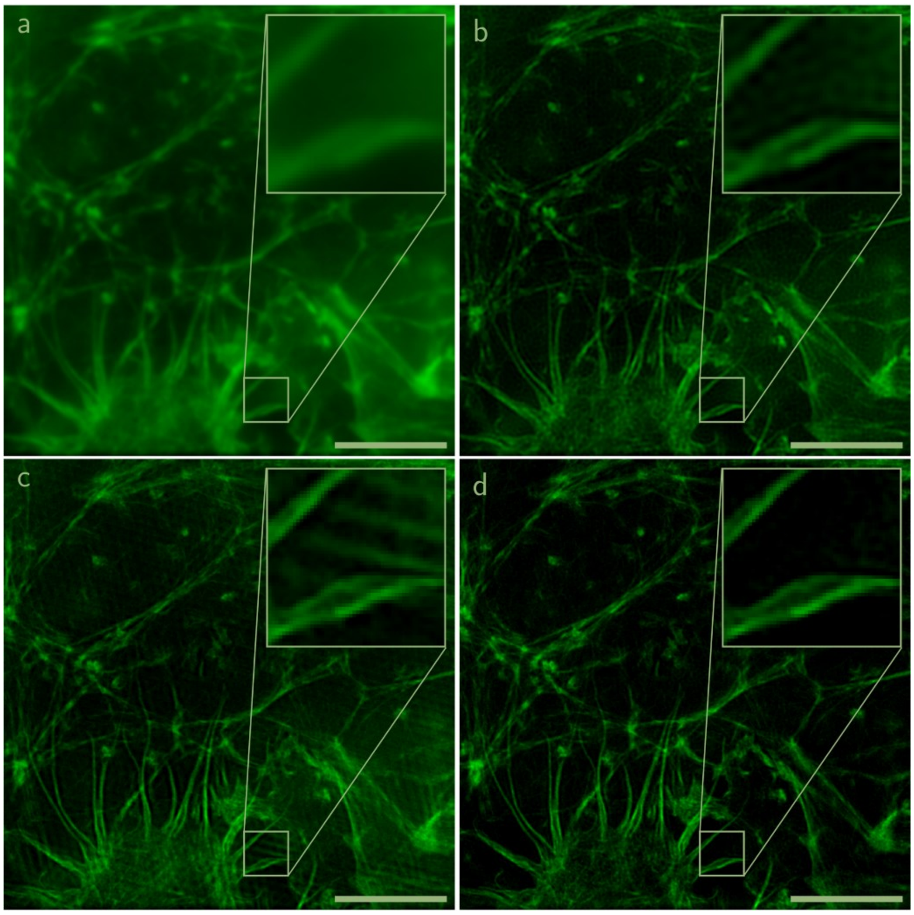

Supplementary Figure 1: Actin filaments in U2OS cells, labelled with Phalloidin-Atto488, measured on a DeltaVision|OMX, excited at 488 nm wavelength. (a) Wide-field, (b) Wiener-filtered wide-field, (c) reconstruction using a standard 2D OTF, (d) reconstruction using the attenuated OTF (strength 0.995, FWHM 2 cycles/micron). Due to the 2D OTFs missing cone problem, background artifacts emerge when reconstructing with an unmodified OTF (c). For 3-beam data (where the bands overlap each others missing cones) and for 2-beam data (trading lateral resolution improvement for a larger overlap) the missing cones can be filled from other bands through the OTF attenuation, and the artifacts are mitigated (d). Thus, the reconstruction gains optical sectioning. Scale bar 5 µm, inset 2.0 µm.

SR-SIM of actin filaments in liver sinusoidal endothelial cells

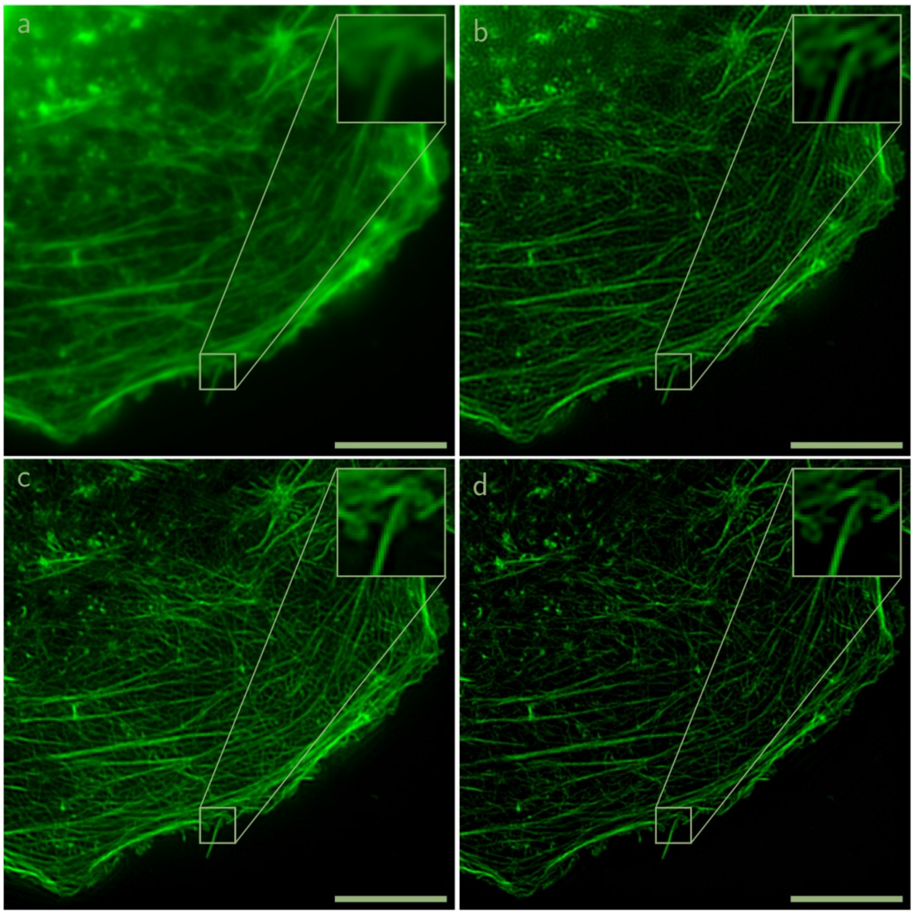

Supplementary Figure 2: Actin filaments in liver sinusoidal endothelial cells (LSECs). DeltaVision|OMX, excited at 488 nm wavelength. (a) Wide-field, (b) Wiener-filtered wide-field, (c) single-slice reconstruction by fairSIM, (d) same slice from full 3D reconstruction by manufacturer software. Note that because the single slice reconstruction in (c) is less restricted in the axial direction, it appears to provide more detail than the 3D reconstruction in (d). This is, however, mitigated e.g. when showing the full 3D data as an intensity projection (not shown). Scale bar 5 µm, inset 1.6 µm.

SR-SIM of U2OS osteosarcoma cells stained for tubulin

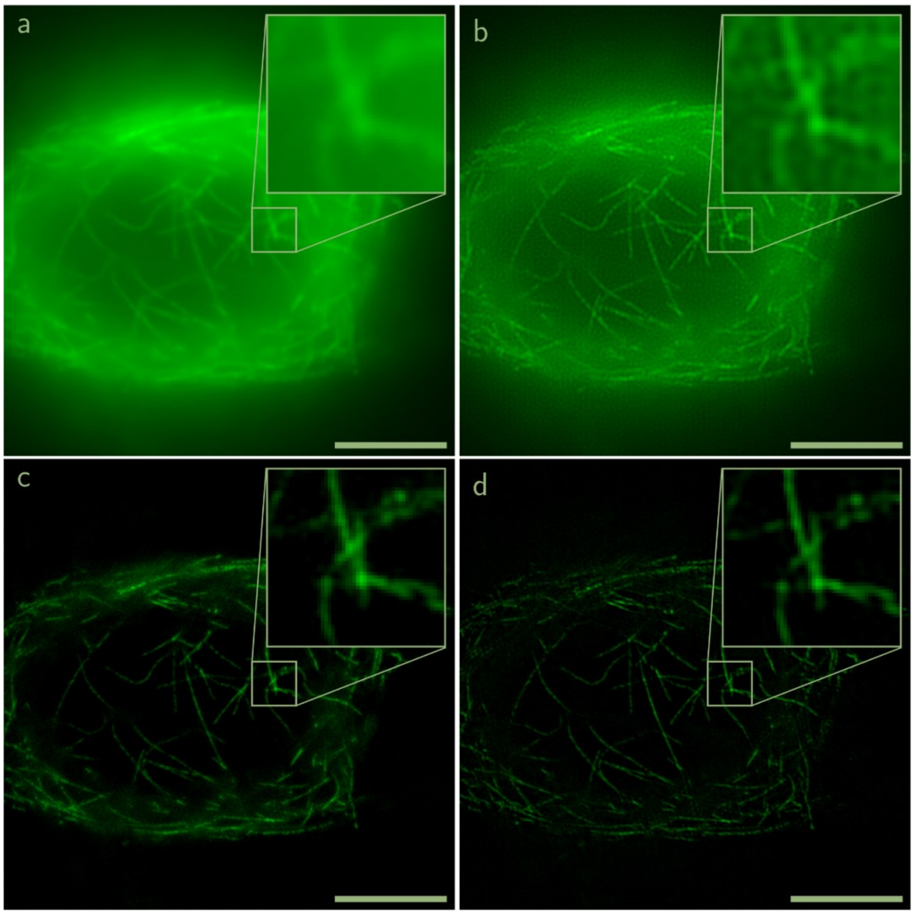

Supplementary Figure 3: Tubulin stain (Alexa 488) in U2OS osteosarcoma cells, DeltaVision|OMX, excited at 488 nm wavelength. (a) Wide-field, (b) Wiener-filtered wide-field, (c) single-slice reconstruction by fairSIM, (d) same slice from full 3D reconstruction by manufacturer software. Note the significant background reduction through the use of optical sectioning (attenuation strength 0.998, FWHM 1.5 cycles/µm). Scale bar 5 µm, inset 2.0 µm.

SR-SIM of mitochondria in U2OS cells

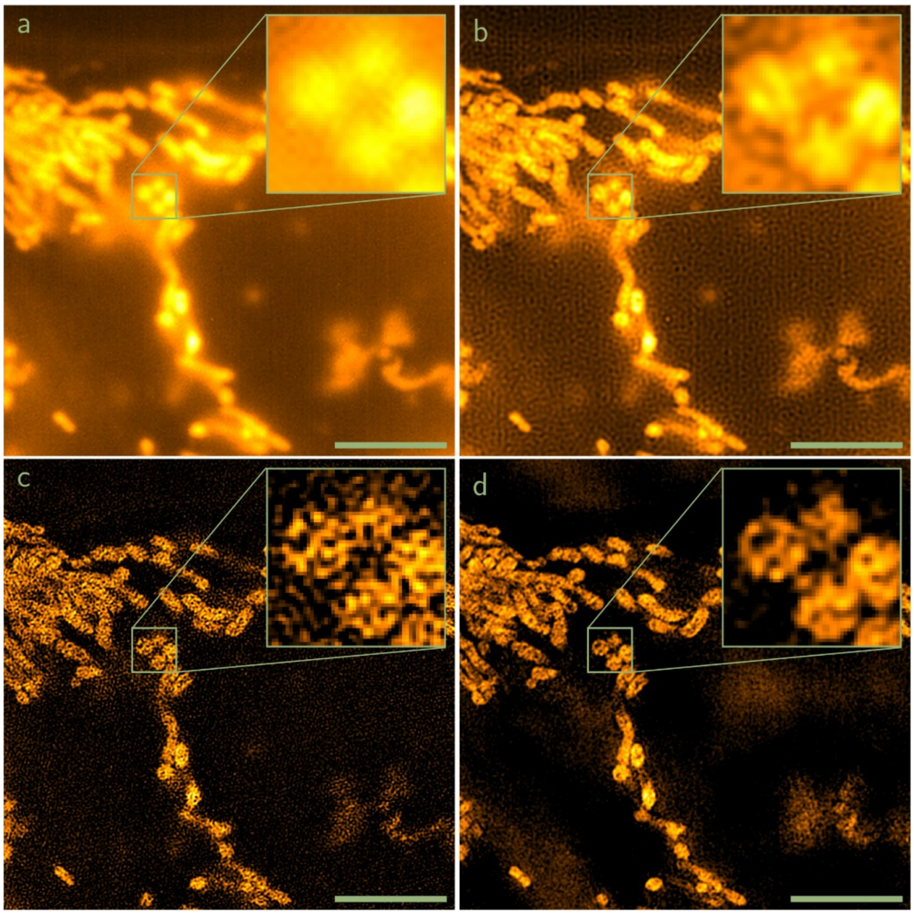

Supplementary Figure 4: Mitrochondria (Mito-Tracker) in U2OS cells, DeltaVision|OMX, excited at 568 nm wavelength. (a) Wide-field, (b) Wiener-filtered wide-field , (c) single-slice reconstruction by fairSIM, (d) same slice from full 3D reconstruction by manufacturer software. Please note, that the single-slice reconstruction suffers from large background and noise levels, but parameter extraction and reconstruction is still possible. Scale bar 5 µm, inset 2.0 µm.

SR-SIM of actin filaments in U2OS cells

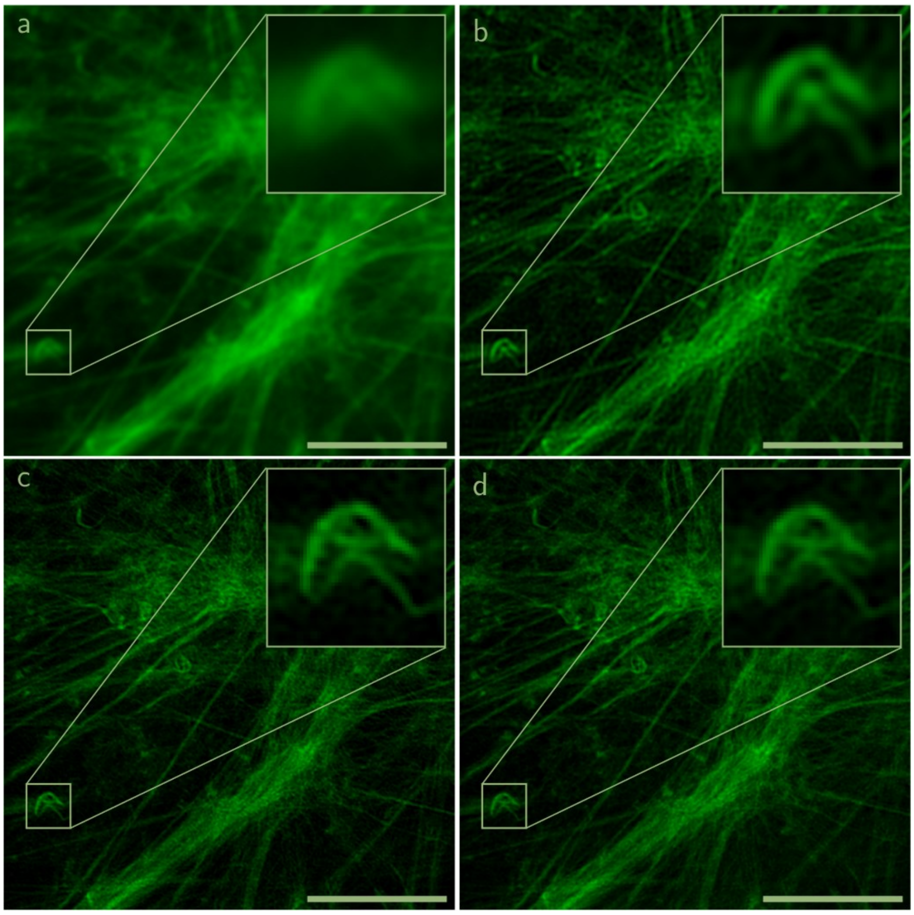

Supplementary Figure 5: Actin filaments in U2OS cells, measured on a Zeiss Elyra S1, excited at 488 nm wavelength. Wide-field, (b) Wiener-filtered wide-field , (c) single-slice reconstruction by fairSIM, (d) same slice from full 3D reconstruction by manufacturer software. Scale bar 5 µm, inset 1.6 µm. Raw datasets[1] courtesy of Marcus Behringer and Markus Sauer, University of Würzburg.

SR-SIM of mitochondria in U2OS cells

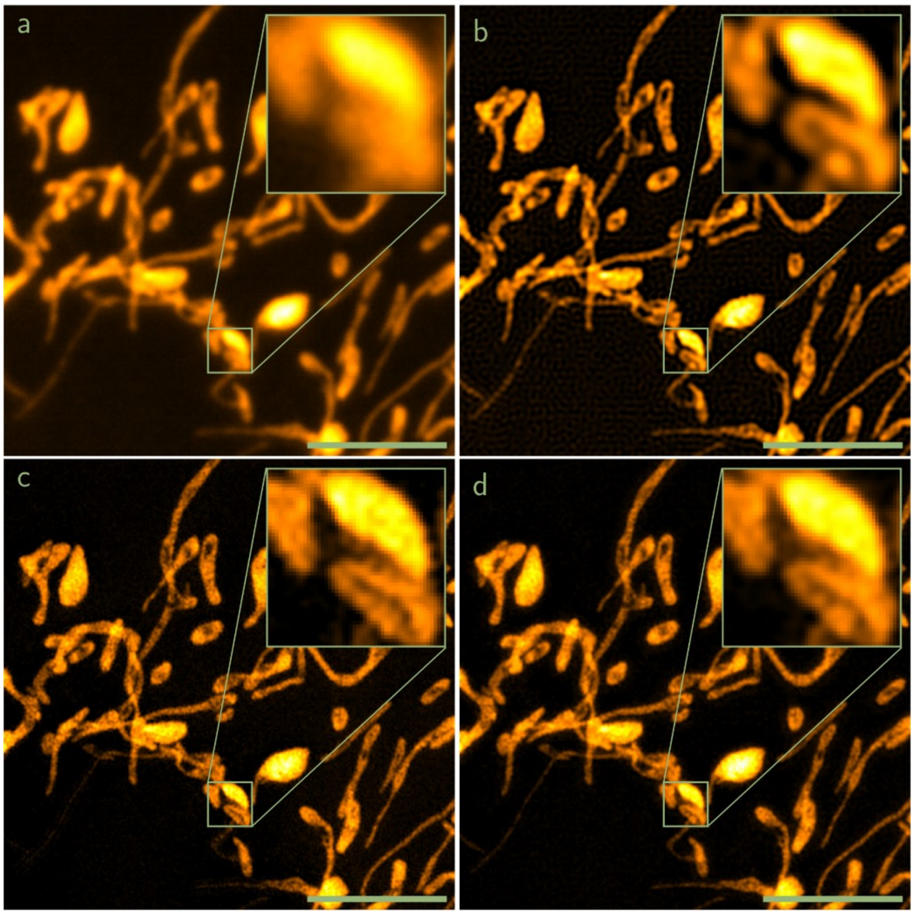

Supplementary Figure 6: Mitochondria in U2OS cells, measured on a Zeiss Elyra S1, excited at 565 nm wavelength. Wide-field, (b) Wiener-filtered wide-field , (c) single-slice reconstruction by fairSIM, (d) same slice from full 3D reconstruction by manufacturer software. Scale bar 5 µm, inset 1.6 µm. Raw datasets[1] courtesy of Marcus Behringer and Markus Sauer, University of Würzburg.

TIRF-SIM of tubulin in cells

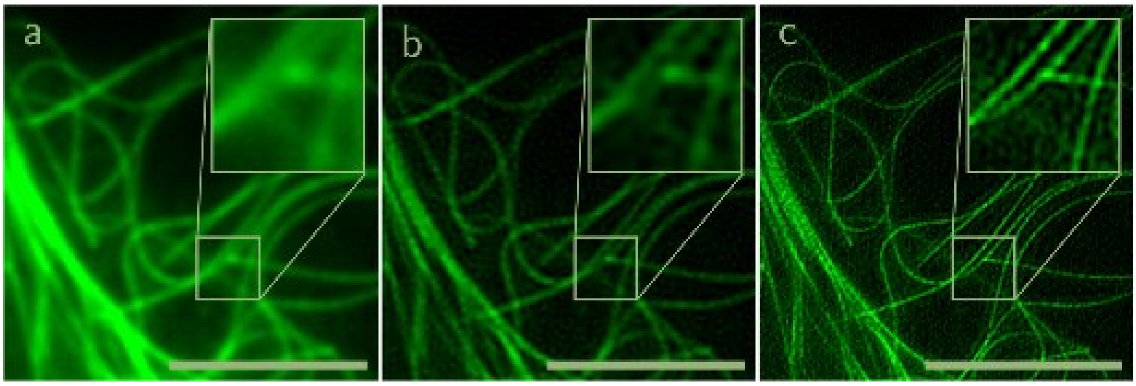

Supplementary Figure 7: Tubulin filaments, measured on a 2D TIRF SIM setup, reconstruction of 9 raw data frames (3 angles, 3 phases), excited at 488nm wavelength. Wide-field, (b) Wiener-filtered wide-field , (c) single-slice reconstruction by fairSIM, which is able to successfully extract the reconstruction parameters from TIRF datasets. Scale bar 5µm, inset 1.6µm. Raw datasets[2, 3] courtesy of Peter Kner, University of Georgia.

Illustration of SR-SIM band overlap

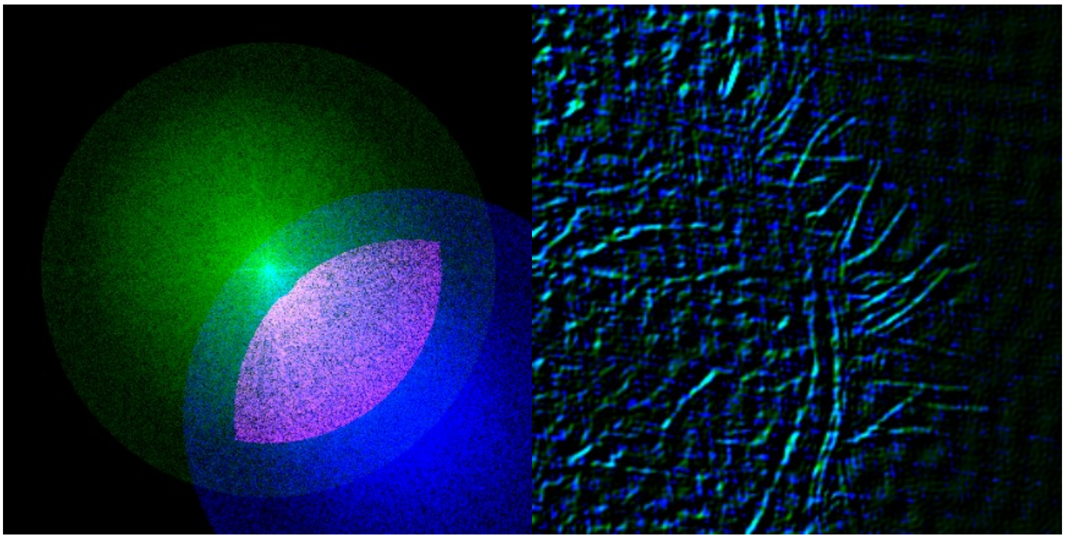

Supplementary Figure 8: Common region of two SIM bands. Left: Power spectra of band $\tilde{S}_0$ (green), shifted band $\tilde{S}_2$ (blue) and the common region (magenta), defined here by $h(\vec{k}), h(\vec{k} + \vec{p}) > 0.05$, i.e. both bands' OTFs are above a certain threshold. Right: Composite spatial representation of only frequencies from the common region, with S_0 (green) and S_2 (blue), base dataset U2OS actin. See Supplementary Note 1 for how the cross-correlation of this band overlap is used in the SR-SIM parameter estimation process.

Supplementary Note 1: Parameter estimation and reconstruction

In super-resolved structured illumination microscopy a sample is illuminated with a series of harmonic illumination intensity patterns. The image sequence $D_n(\vec{r})$ acquired by the microscope is given by[1]

$$D_n(\vec{r}) = \sum_{m=-M}^{M} [S(\vec{r})\, a_m \exp\{im(2\pi\vec{p}\vec{r} + \phi_n)\}] \otimes \tilde{h}(\vec{r}).$$

Here $S(\vec{r})$ denotes the fluorophores' response to light, i.e. the property to be measured. M denotes the number of harmonics, so $M = 1$ for two-beam, and $M = 2$ for three-beam illumination. $\vec{p}$ is the modulation light wave vector, i.e. the pattern orientation and spacing. ϕ_n denotes the phase, a_m the modulation depth of the pattern. $\tilde{h}(\vec{r})$ is the point-spread function, which causes the resolution limit. Switching to Fourier space, each harmonic illumination pattern transforms to delta-peaks at $\delta(\pm m\vec{p})$ and, using their folding and translation properties

$$\tilde{D}_n(\vec{k}) = \sum_{m=-M}^{M} \exp\{im\phi_n\}\, a_m\, \tilde{S}(\vec{k} - m\vec{p}) \cdot h(\vec{k})$$

is obtained. The translation of $\tilde{S}$ to $\vec{k} - m\vec{p}$, caused by the delta-peaks' position at $\delta(m\vec{p})$, is what allows previously unobservable frequencies in $\tilde{S}$ to move into the support of $h(\vec{k})$, thus yielding the resolution improvement in SIM. The component $\tilde{S}_m(\vec{k}) = S(\vec{k} - m\vec{p})$ is referred to as the m'th band. Bands are symmetric by construction, i.e. S_{-m} contains the same information as S_m. Now, acquiring N measurements of $D_n(\vec{k})$, with varying (usually equi-distant) phases ϕ_n, but fixed $\vec{p}$, allows to rewrite the equation to matrix form as

$$\tilde{\boldsymbol{D}}(\vec{k}) = \boldsymbol{M}\boldsymbol{C}(\vec{k})$$

with vectors

$$\begin{aligned}\tilde{\boldsymbol{D}}(\vec{k}) &= [\,D_1(\vec{k}), \dots, D_N(\vec{k})],\\ \boldsymbol{C}(\vec{k}) &= [\,C_{-M}(\vec{k}), \dots, C_0(\vec{k}), \dots, C_M(\vec{k})]\\ &= [C(\vec{k} + m\vec{p}), \dots, C(\vec{k}), \dots, C(\vec{k} - m\vec{p})]\end{aligned}$$

and where

$$\boldsymbol{M}_{nm} = a_m \exp im\phi_n \quad \text{and} \quad C(\vec{k}) = \tilde{S}(\vec{k})\, h(\vec{k})$$

With a sufficient number ($N \geq M$) of known phases ϕ_n, $\boldsymbol{M}$ can be inverted[2], so with

$$\boldsymbol{C}(\vec{k}) = \boldsymbol{M}^{-1}\, \tilde{\boldsymbol{D}}(\vec{k})$$

all bands $\tilde{S}_m(\vec{k})$ in $\boldsymbol{C}(\vec{k})$ can be extracted. This step is referred to as *band separation*.

---

[1] Mathematical naming conventions largely follow the ones used by Heintzmann et. al.

[2] For an over-defined matrix ($N > M$), the Moore-Penrose pseudo-inverse is used to invert $\boldsymbol{M}$.

Afterwards, the bands $\tilde{S}_m(\vec{k})$ are moved to their correct position $\pm m\vec{p}$ in Fourier space, added up and transformed back to a high resolution image in real space. The image assembly in Fourier space is usually performed through a Wiener filter:

$$\tilde{S}_{SR}(\vec{k}) = \frac{\sum_{m=-M}^{M} h^*(\vec{k} + m\vec{p}) \cdot C_m(\vec{k} + m\vec{p})}{\omega^2 + \sum_{m=-M}^{M} h^2(\vec{k} + m\vec{p})} A(\vec{k})$$

The filtering compensates for the frequency dampening introduced by $h(\vec{k})$, the parameter ω dampens the degree of compensation especially in regions where h is low. It should thus be set in accordance to the SNR of the input data. $A(\vec{k})$ is the apodization, compensating for ringing artifacts.

A SIM reconstruction thus amounts to Fourier-transforming the input, carrying out band-separation, shifting the bands to $m\vec{p}$, summing them up through (e.g.) a Wiener filter, and transforming the result back to a high resolution image. The resolution gain is given by the length $|M\vec{p}|$, which is approximately[3] limited to the same cut-off as $h(\vec{k})$ for linear SIM[4]. Thus, SIM typically doubles the resolution in comparison to a wide-field measurement.

Parameter estimation:

The SIM reconstruction introduced so far needs correct parameters (pattern spacing and orientation $\vec{p}$, phases ϕ_n) to be carried out, which are typically[5] extracted from the input data. A reliable algorithm to obtain this estimation is often much more involved than the reconstruction itself. The method employed be fairSIM follows the method by Gustaffson et. al.[4]. Because the original publication provides little detail on the parameter estimation, we briefly lay out the mathematical background here. In general, the use of cross- and autocorrelation of frequency components for SIM parameter estimation is documented in a number of recent publications[4, 5, 6].

Assuming equi-distant phases ϕ'_m , differing from correct phases $\phi_m = \phi'_m + \phi_\Delta$ only by a global offset ϕ_Δ, the band-separation step is carried out.

For linear SIM[6], the separated and OTF-corrected bands $\tilde{S}_m$ will have common, overlapping regions (see Supplementary Fig. 8 for an illustration).With $\vec{k}'$ denoting only components within these regions, $\tilde{S}_m(\vec{k}' + m\vec{p})$ and $\tilde{S}_0(\vec{k}')$ should only differ by a constant, complex factor a'_m. As

$$\tilde{S}_m(\vec{k}' + m\vec{p}) = a'_m \cdot \tilde{S}_0(\vec{k}')$$

only holds for the correct shift vector $\vec{p}$, the cross-correlation

---

[3] For an exact number, wavelength change (Stokes-shift) has to be taken into account, and TIRF illumination will further shift the limit somewhat.

[4] Non-linear SIM completely circumvents the limit by using effects such as photo-switching, depletion, or two-photon excitation.

[5] Stable systems, especially SLM-based, should yield rather constant reconstruction parameters over time. However, retrieving a parameter estimate from data should often be much easier than characterizing them from the experimental properties. It might then be used to run multiple reconstructions.

[6] For non-linear SIM, at least neighboring bands S_{m-1}, S_m will overlap (otherwise, frequencies are missing from the reconstruction), thus the process can be carried out iteratively.

$$\mathcal{C}(\vec{p}) = \left[\,\tilde{S}_m \star \tilde{S}_0\,\right](\vec{p}) = \sum_{\vec{k}'} \tilde{S}_m^*\big(\vec{k}' + m\vec{p}\big)\,\tilde{S}_0(\vec{k}')$$

again with summation $\sum_{\vec{k}'}(\ldots)$ limited to a region common to both band, will reach a maximum at the correct $\vec{p}$. This can be used in an iterative search to find $\vec{p}$, and implemented effectively, to sub-pixel precision, via the Fourier shift theorem. FairSIM provides visual feedback of this fit process, so the user can check it for plausibility.

Now, at the correct $\vec{p}$, the equivalence given above holds, so a'_m is found as

$$a'_m = \frac{\mathcal{C}(\vec{p})}{\left|\tilde{S}_0^2\right|_{\vec{k}'}} = \frac{\sum_{\vec{k}'} \tilde{S}_m^*\big(\vec{k}' + m\vec{p}\big)\,\tilde{S}_0(\vec{k}')}{\sum_{\vec{k}'} \tilde{S}_0^2(\vec{k}')}$$

and yields both, the global phase offset $\phi_\Delta = \arg(a')$ and – in principle – the pattern modulation depth $a_m = |a'|$.

Supplementary References:

1. Sauer M. Raw SIM images Zeiss Elyra S1. (personal communication Huser T.) (2015).

2. Kner P. Raw datsets TIRF SIM. (personal communication Huser T.) (2015).

3. Kner P, Chhun BB, Griffis ER, Winoto L, Gustafsson MGL. Super-resolution video microscopy of live cells by structured illumination. *Nat Methods* **6**, 339-342 (2009).

4. Gustafsson MGL*, et al.* Three-dimensional resolution doubling in wide-field fluorescence microscopy by structured illumination. *Biophys J* **94**, 4957-4970 (2008).

5. Wicker K. Non-iterative determination of pattern phase in structured illumination microscopy using auto-correlations in Fourier space. *Opt Express* **21**, 24692-24701 (2013).

6. Wicker K, Mandula O, Best G, Fiolka R, Heintzmann R. Phase optimisation for structured illumination microscopy. *Opt Express* **21**, 2032-2049 (2013).
